# Pharmacodynamic effects of direct AMP kinase activation in humans with insulin resistance and non-alcoholic fatty liver disease: A phase 1b study

**DOI:** 10.1016/j.xcrm.2021.100474

**Published:** 2021-12-21

**Authors:** Pascale Fouqueray, Sebastien Bolze, Julie Dubourg, Sophie Hallakou-Bozec, Pierre Theurey, Jean-Marie Grouin, Clémence Chevalier, Pascale Gluais-Dagorn, David E. Moller, Kenneth Cusi

**Affiliations:** 1 Poxel SA, Lyon, Auvergne-Rhône-Alpes, France; 2 University of Rouen, Rouen, Normandie, France; 3 Division of Endocrinology, Diabetes and Metabolism, University of Florida, Gainesville, FL, USA

**Keywords:** AMPK activation, non-alcoholic fatty liver disease, NAFLD, non-alcoholic steatohepatitis, NASH, metabolic syndrome-associated fatty liver disease, MAFLD, de novo lipogenesis, DNL, insulin resistance, PXL770, pharmacokinetics, pharmacodynamics, plasma lipids

## Abstract

AMPK is an energy sensor modulating metabolism, inflammation, and a target for metabolic disorders. Metabolic dysfunction results in lower AMPK activity. PXL770 is a direct AMPK activator, inhibiting *de novo* lipogenesis (DNL) and producing efficacy in preclinical models. We aimed to assess pharmacokinetics, safety, and pharmacodynamics of PXL770 in humans with metabolic syndrome-associated fatty liver disease. In a randomized, double-blind four-week trial, 12 overweight/obese patients with non-alcoholic fatty liver disease (NAFLD) and insulin resistance received PXL770 500 mg QD; 4 subjects received matching placebo. Endpoints included pharmacokinetics, hepatic fractional DNL, oral glucose tolerance testing, additional pharmacodynamic parameters, and safety. PK parameters show adequate plasma exposure in NAFLD patients for daily oral dosing. PXL770 decreases DNL—both peak and AUC are reduced versus baseline—and improves glycemic parameters and indices of insulin sensitivity versus baseline. Assessment of specific lipids reveals decrease in diacyglycerols/triacylglycerols. Safety/tolerability are similar to placebo. These results unveil initial human translation of AMPK activation and support this therapeutic strategy for metabolic disorders.

## Highlights

• AMPK is impaired in metabolic diseases—PXL770 is a direct AMPK activator
• In humans with NAFLD, PXL770 inhibits hepatic *de novo* lipogenesis (target engagement)
• PXL770 also improves glucose tolerance and insulin sensitivity
• PXL770 reduces plasma levels of di- and triglycerides

## Graphical abstract

**Figure:**
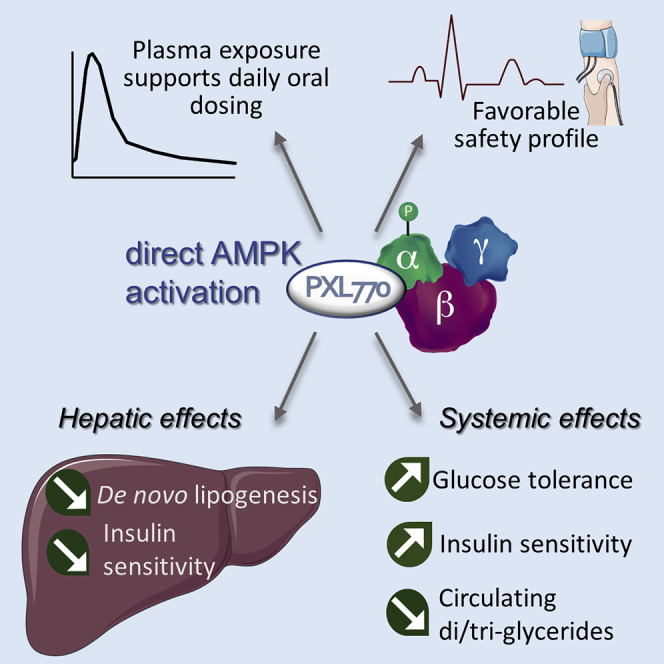

## Teaser

AMPK is a bioenergetic sensor modulating metabolism/inflammation, with reduced activity in metabolic diseases. PXL770 is a direct AMPK activator investigated for non-alcoholic steatohepatitis and adrenoleukodystrophy. In this clinical study, Fouqueray and colleagues demonstrate that PXL70 inhibits hepatic lipogenesis and improves glucose metabolism and insulin sensitivity in non-alcoholic fatty liver disease patients.

## Introduction

AMP activated protein kinase (AMPK) is a major regulator of cellular energy metabolism with orthologs that have been identified in all eukaryotic species. In mammals, AMPK is a heterotrimeric enzyme complex consisting of distinct subunits (α, β, γ); it is primarily activated by upstream kinases in response to an increase AMP/ADP relative to ATP.^1^^,^^2^ In general, AMPK inhibits selected biosynthetic pathways while activating catabolism to sustain energy availability in the face of decreased energy intake or increased expenditure.^2^ Therefore, AMPK is activated in response to physiological stimuli including fasting and exercise.

AMPK activation modulates pleiotropic metabolic pathways that lead to stimulation of lipid consumption (fatty acid oxidation), increased glucose uptake, and enhanced aerobic metabolism through mitochondria biogenesis.^1^^,^^2^ It also has a major role to inhibit *de novo* lipogenesis (DNL) via direct phosphorylation of acetyl CoA carboxylase (ACC). A potential effect to suppress lipolysis in adipocytes has also been described; however, this finding remains somewhat controversial.^3^^,^^4^ Additional studies have implicated a role for AMPK activation in enhancing insulin sensitivity per se.^5^^,^^6^

Beyond effects on metabolic pathways, AMPK activation has also been shown to inhibit multiple pro-inflammatory pathways and to activate the secretion of anti-inflammatory cytokines.^7^ In the context of experiments focusing on the pathophysiology of non-alcoholic steatohepatitis (NASH), AMPK activation has anti-fibrotic effects including inhibition of hepatic stellate cell activation and the production of extracellular matrix;^8^ an additional important effect to attenuate disease-related hepatocellular apoptosis was also described.^9^

Given the aforementioned effects of AMPK activation, which have been demonstrated in a variety of cell-based and *in vivo* preclinical studies, this approach has been proposed as a therapeutic option to target numerous diseases across a broad spectrum of metabolic disorders such as type 2 diabetes (T2D), NASH, obesity, metabolic syndrome, and diabetic kidney disease^10^ as well as rare diseases characterized by defective lipid metabolism such as adrenoleukodystrophy^11^ and other orphan indications including autosomal dominant polycystic kidney disease.^12^ Other instances where AMPK has been commonly implicated as a therapeutic strategy are neurodegeneration and certain forms of cancer, in particular, hepatocellular carcinoma.^10^

Non-alcoholic fatty liver disease (NAFLD) and its more severe form, NASH, have been frequently emphasized as preferred therapeutic targets for AMPK.^13^ Around one-fourth of the worldwide adult population suffers from NAFLD^14^ and its progression ultimately results in cirrhosis, hepatocellular carcinoma, and other adverse sequelae. Furthermore, no approved pharmacologic treatments for NAFLD-NASH are available today.^14^^,^^15^ Of particular note, increased hepatic DNL is a substantial contributor to steatosis and has been estimated to account for up to 38% of excess liver fat content in obese NAFLD patients.^16^ Importantly, the onset and progression of NAFLD-NASH are coupled with insulin resistance through several overlapping pathways involving tissue lipid accumulation, inflammation, cellular stress, and mitochondrial dysfunction.^17^ Thus, therapies that augment insulin sensitivity have been proposed as approaches to NAFLD-NASH.^15^^,^^18^ Accordingly, T2D is also the most important co-morbidity in patients with NAFLD or NASH, where over 45% of NAFLD-NASH patients also suffer from T2D.^19^^,^^20^ The close association between metabolic dysfunction and NAFLD pathophysiology has led experts to propose a change in nomenclature: metabolic dysfunction-associated fatty liver disease, or MAFLD.^21^^,^^22^

The ability to activate AMPK with pharmacologic agents was enabled by the recent discovery of several small molecule allosteric activators; these compounds appear to bind to a common pocket at the α and β subunit interface known as the allosteric drug and metabolism (ADaM) site which also involves the β subunit carbohydrate binding module (CBM).^10^ Despite significant efforts, no previously published reports have shown the effects of other direct AMPK activators in humans.

PXL770 is a small molecule that directly activates human AMPK *in vitro* with an allosteric mechanism mediated by binding to the AdaM site. In animal models of NASH and T2D it attenuates several features of liver pathology, reverses hyperglycemia, and enhances insulin sensitivity; in addition, *in vitro* effects to suppress DNL (hepatocytes), attenuate inflammation (macrophages), or to potentially reduce fibrosis (stellate cells) have been observed in human cell-based assays.^23^

Here we studied the effects of daily oral administration of PXL770 in overweight or obese human subjects with NAFLD and insulin resistance for 4 weeks. This randomized, double-blind parallel design trial met its primary endpoints, allowing for full characterization of the pharmacokinetic profile in this population. Preliminary safety was evaluated and early signals of efficacy including target engagement in the liver were observed in this study.

## Results

### Subject demographics and disposition

**Figure 1:**
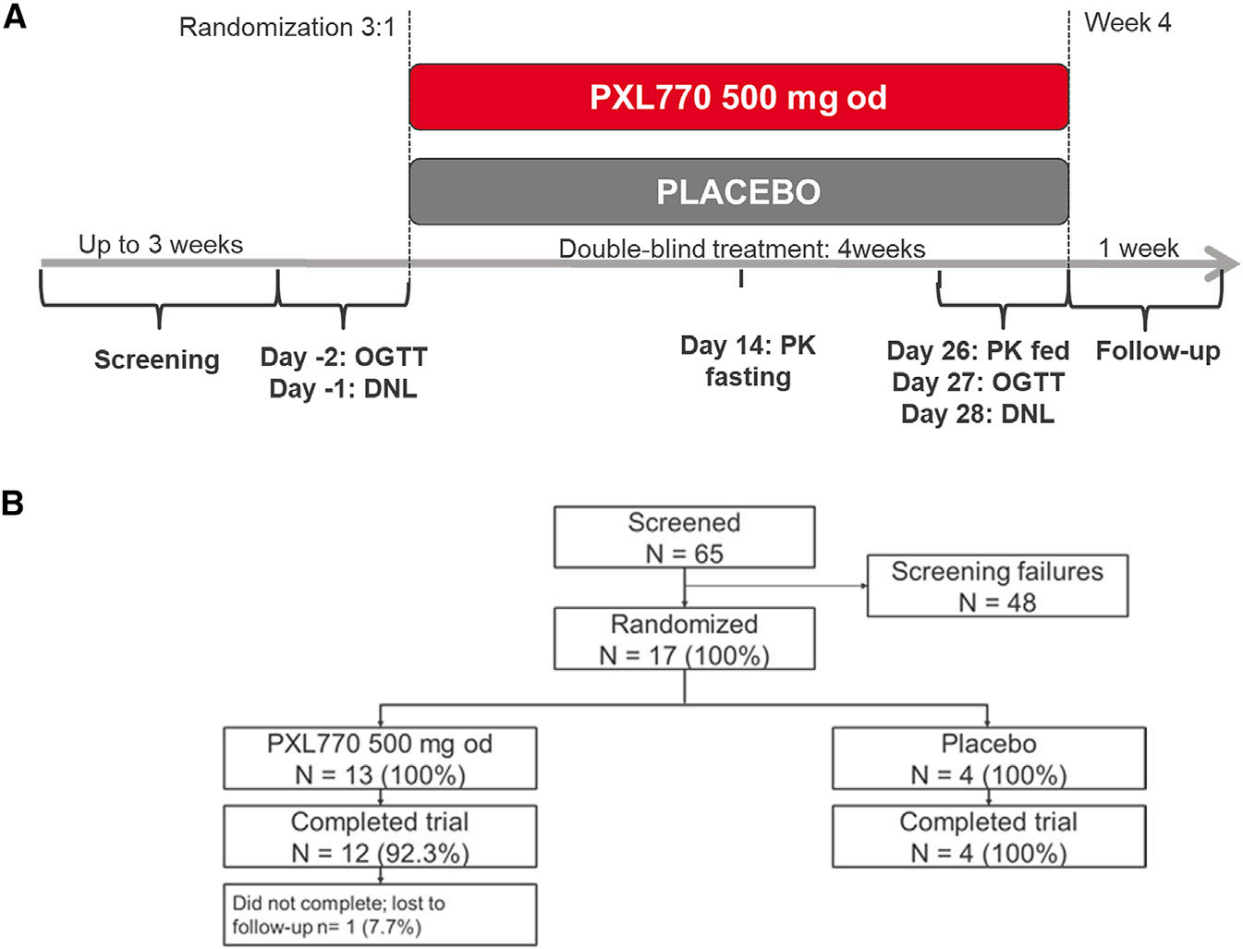
Study design and patient disposition

The study design is presented in Figure 1A; the study was registered with ClinicalTrials.gov: NCT03950882. Subjects were screened between August 1, 2019, and March 12, 2020. Of the 65 subjects screened, 17 were considered eligible and were randomly assigned to receive 500 mg PXL770 qd (n = 13) or placebo (n = 4, Figure 1B). The most common reason for screen failure was not meeting inclusion criteria for hepatic steatosis or for ALT. The small number of placebo subjects were included in the study for safety evaluation purposes and as a control for possible study effects. All patients received study drug and completed the study, except one who was lost to follow up. Further details regarding trial design, inclusion criteria, and implementation are described in StarMethods.

The baseline characteristics of the subjects are presented in Table S1. Most patients were female (64.7%), white (76.5%), and not Hispanic or Latino (58.8%). The mean age was 47.5 years. All patients were overweight or obese, and the me an BMI was 37.7 kg/m^2^. Mean baseline CAP score measured by transient elastography (Fibroscan) was 350 dB/m, indicating a substantial degree of hepatic steatosis.^24^ Mean baseline fasting plasma glucose (FPG) was 110.4 mg/dL; mean baseline HOMA-IR (homeostatic model of insulin resistance) values were greater than 8.5; values of < 2.6 are generally considered to be in the normal range.^25^

### Pharmacokinetics

**Figure 2:**
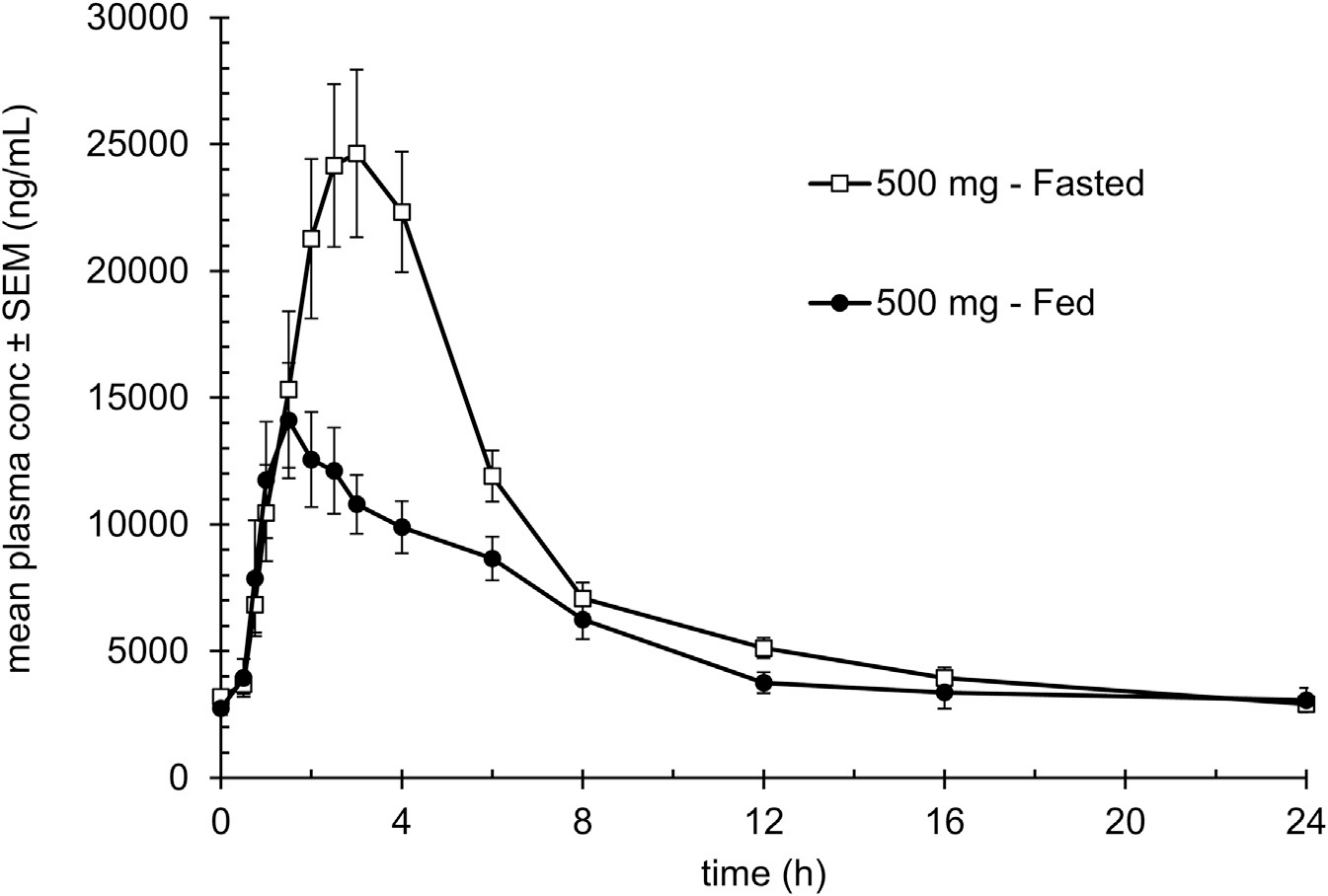
Mean plasma levels of PXL770 Steady-state plasma samples were obtained from subjects (n = 12 patients) receiving daily oral doses of 500 mg PXL770 on two occasions during the study, day 14 and day 26 (Figure 1), under fasted and fed conditions, respectively. Samples were analyzed to determine drug concentrations using a validated liquid chromatography with tandem mass spectrometry (LC-MS/MS) method.

Pharmacokinetic measurements were performed on two occasions: days 14 (fasted) and 26 (fed where test article dosing occurred 15 min before a standardized breakfast). In both the fasted and fed states, PXL770 was rapidly absorbed and distributed. The maximal plasma concentration of PXL770 was reached at a median of 2.25 and 2.75 h post-dose in the fasted and fed state, respectively (Figure 2). Concentrations of PXL770 then declined in a biphasic manner with a rapid first distribution-elimination phase followed by a long terminal elimination phase, with a geometric mean t¾¾¾ of 18.7 h in the fed state. Mean plasma concentrations were also moderately lower when measured in the fed state compared with fasting values. The geomean C_max_ was 37% lower (16.1 μg/mL) under fed versus fasted (25.5 μg/mL) conditions; similarly, the geomean AUC_0-24 h_ was 29% lower (130.8 μg.h/mL) in the fed versus fasted (183.1 μg.h/mL) state. Geomean trough plasma levels were approximately 10%–15% of geomean C_max_ values (Figure 2). Thus, a significant negative food effect was evident from these data when PXL770 was administered as a capsule formulation. The PK profile observed in these NAFLD patients was comparable to the one observed previously in healthy volunteers including the negative food effect (data not shown). Importantly, the average exposure (AUC) achieved under fed conditions (conditions of drug intake throughout the 28 day study, with the exception of OGTT or DNL test days, was within or modestly above plasma exposure levels that were associated with efficacious doses (NASH or T2D models) in mice (approximately 80-100 μg.h/mL; data not shown).

### Pharmacodynamics (PD)

Several parameters designed to assess target engagement and potential efficacy signals were assessed. Since only four placebo-treated subjects were included in the study (primarily for safety comparison purposes), PD analyses were mainly focused on comparisons of baseline measurements to the end of the treatment period in the PXL770 treated subjects.

**Figure 3:**
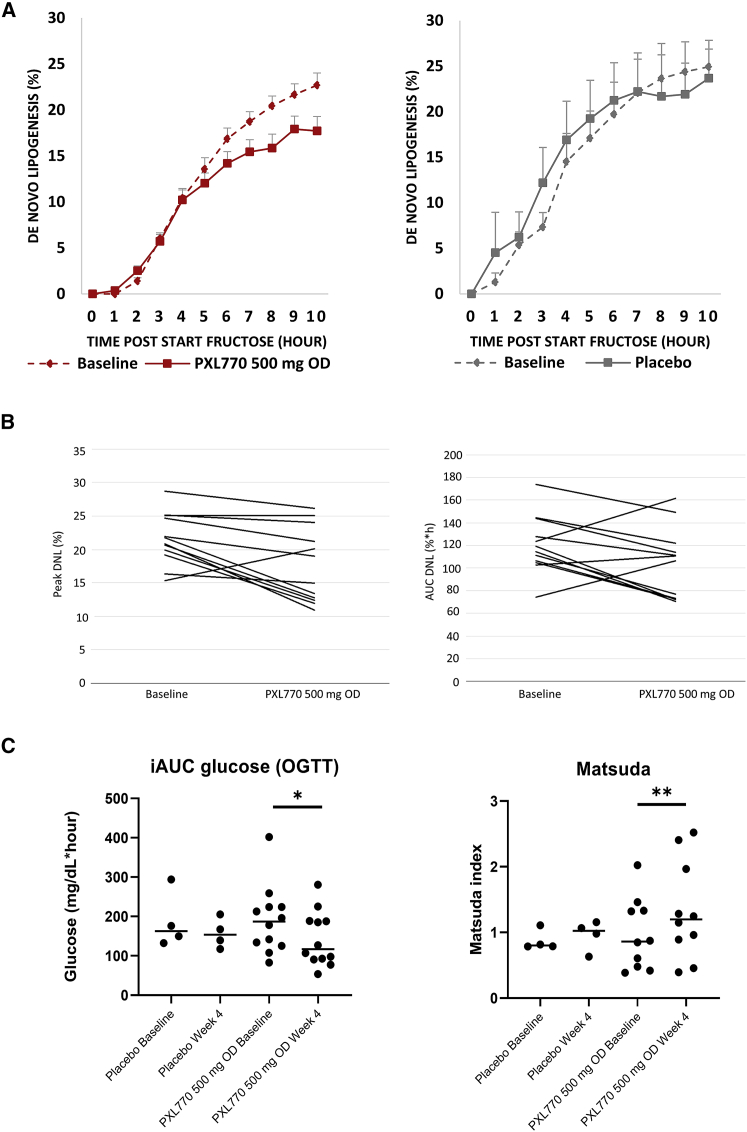
PXL770 inhibits *de novo* lipogenesis (DNL) and improves glucose metabolism and insulin sensitivity DNL was measured as described in StarMethods by labeling of body water via D_2_0 enrichment during the day prior to testing followed by oral fructose loading during the test days −1 and 28 (Figure 1). Fractional DNL (% of triglyceride-palmitate) was calculated based on measurements obtained at the indicated time points. Oral glucose tolerance tests were performed after an overnight fast at baseline and on day 27 during treatment. (A) Time course of mean (+SEM) DNL values for PXL770 (left panel; n = 12, p = 0.0045 for peak DNL, defined as average of last 3 time points) or placebo (right panel; n = 4, NS) treated subjects at baseline (dashed line) versus end of treatment on day 28 (solid line). (B) Individual patient results for DNL tests performed at baseline versus after 28 days of daily exposure to PXL770 (n = 12 patients). (C) Mean and individual values for incremental glucose AUC during the oral glucose tolerance test, OGTT (n = 4 placebo, n = 12 PXL770), and for the Matsuda insulin sensitivity index, data derived from the OGTT (n = 4 placebo, n = 10 PXL770), in subjects treated with placebo or PXL770 at baseline versus day 27. Matsuda values were calculated based on data obtained during OGTT tests as described in StarMethods. ∗p = 0.031; ∗∗p = 0.014.

Fractional hepatic DNL was measured at baseline (day −1) and on day 28, using well-established and validated methods,^26^ by first exposing subjects to D_2_O followed by maximal DNL stimulation via oral ingestion of excess fructose after an overnight fast. The time course of DNL in PXL770 and placebo groups are shown in Figure 3A. The mean AUC (during the 10 h test period) showed a trend for DNL to decrease from baseline to week 4 by 17.45%∗h in PXL770 subjects (Figure 3B, p = 0.074) in the mITT analysis. This difference was significant (−1.32%∗h, p = 0.031) in an additional analysis of the PPS population where two subjects were not included (one was treated for 19 days relating to a COVID issue; a second subject developed elevated FPG levels after screening). Peak DNL was significantly decreased by 4.57%∗h (−20% relative change to baseline) after 4 weeks of treatment in the PXL770 subjects (Figure 3B, p = 0.0045). There was no difference in DNL AUC or peak levels between baseline and week 4 in the placebo group (Figure 3A). These results indicate a significant effect of PXL770 to suppress DNL and are consistent with the known effects of AMPK to decrease DNL via phosphorylation of acetyl CoA carboxylase (ACC).^13^

Standard glucose tolerance tests (OGTT) were performed at baseline (day −2) and on day 27. Relative to measurements obtained at baseline, mean total AUC (Table 1) and incremental (iAUC, Figure 3C) glucose excursions were significantly reduced after 4 weeks of daily treatment with PXL770 (p = 0.023 and p = 0.031). FPG was also significantly reduced (p = 0.0014, Table 1). However, there was no evidence of potential changes in the total AUC and iAUC for insulin, C-peptide, and free fatty acids (FFA) (Table 1). The potential effects on FPG or glucose tolerance with PXL770 were not significant when compared to placebo; there also was a similar (but not significant) trend toward improvements in FPG and AUC glucose during the OGTT in placebo subjects. HOMA-IR,^25^ an index predominantly of hepatic insulin sensitivity based on fasting glucose and insulin, was improved in PXL770-treated subjects versus baseline but not versus placebo (Table 1). Calculated indices of insulin sensitivity that were derived from the OGTT data, Matsuda^27^ and OGIS,^28^ were significantly improved (Figure 3D and Table 1; p = 0.014 and p = 0.012, respectively, versus baseline). The effect on OGIS was also significant versus placebo (p = 0.028). Within the placebo group, there were no changes from baseline in insulin sensitivity indices.

**Table 1:** Additional pharmacodynamic measurements in subjects exposed to PXL770 for 4 weeks

	Parameter	PXL770 (n = 12)	Placebo (n = 4)
**FPG (mg/dL)**			

Baseline	mean	115.0	94.5
Change from baseline	LS mean	−11.0	−8.4
	SE of LS mean	2.7	4.7
	p value versus baseline	0.0014	0.10
	p value versus placebo	0.64	

**AUC glucose (mg/dL∗h)**			

Baseline	mean	523.5	462.2
Change from baseline	LS mean	−45.5	−51.8
	SE of LS mean	17.4	30.4
	p value versus baseline	0.023	0.11
	p value versus placebo	0.86	

**AUC C-peptide (ng∗h/mL)**			

Baseline	Mean	40.9	45.1
Change from baseline	LS mean	−2.0	−3.9
	SE of LS mean	2.5	4.3
	p value versus baseline	0.44	0.38
	p value versus placebo	0.71	

**iAUC C-peptide (ng∗h/mL)**			

Baseline	Mean	25.2	30.2
Change from baseline	LS mean	−1.7	−3.4
	SE of LS mean	2.4	4.2
	p value versus baseline	0.50	0.44
	p value versus placebo	0.73	

**AUC insulin (μU∗h/mL)**			

Baseline	Mean	674.1∗	841.3
Change from baseline	LS mean	−24.7	−57.6
	SE of LS mean	82.7	138.2
	p value versus baseline	0.77	0.68
	p value versus placebo	0.84	

**iAUC insulin (μU∗h/mL)**			

Baseline	Mean	548.7∗	735.4
Change from baseline	LS mean	−18.8	−62.2
	SE of LS mean	82.7	138.8
	p value versus baseline	0.82	0.66
	p value versus placebo	0.80	

**AUC FFA (mmol∗h/L)**			

Baseline	mean	0.73	0.53
Change from baseline	LS mean	0.04	−0.05
	SE of LS mean	0.07	0.12
	p value versus baseline	0.55	0.68
	p value versus placebo	0.52	

**Matsuda**			

Baseline	mean	0.97∗	0.87
Change from baseline	LS mean	0.37	0.10
	SE of LS mean	0.12	0.19
	p value versus baseline	0.014	0.62
	p value versus placebo	0.27	

**OGIS**			

Baseline	mean	224.3∗	252.7
Change from baseline	LS mean	37.6	−21.8
	SE of LS mean	12.3	19.4
	p value versus baseline	0.012	0.29
	p value versus placebo	0.028	

**HOMA-IR**			

Baseline	mean	15.4	8.8
Change from baseline	LS mean	−4.3	−2.2
	SE of LS mean	1.5	2.6
	p value versus baseline	0.013	0.42
	p value versus placebo	0.50	

**TG (mg/dL)**			

Baseline	mean	151.2	179.5
Change from baseline	LS mean	2.9	9.5
	SE of LS mean	11.9	20.9
	p value versus baseline	0.81	0.66
	p value versus placebo	0.79	

**VLDL cholesterol (mg/dL)**			

Baseline	mean	30.3	35.8
Change from baseline	LS mean	0.6	1.8
	SE of LS mean	2.4	4.2
	p value versus baseline	0.82	0.68
	p value versus placebo	0.81	

**Apolipoprotein B (mg/dL)**			

Baseline	mean	102.0	95.5
Change from baseline	LS mean	3.2	−4.3
	SE of LS mean	4.1	7.1
	p value versus baseline	0.45	0.55
	p value versus placebo	0.38	

**HDL-cholesterol (mg/dL)**			

Baseline	mean	43.8	40.3
Change from baseline	LS mean	−2.0	−1.7
	SE of LS mean	1.2	2.1
	p value versus baseline	0.11	0.42
	p value versus placebo	0.91	

**LDL-cholesterol (mg/dL)**			

Baseline	mean	118.9	102.0
Change from baseline	LS mean	0.2	−6.1
	SE of LS mean	5.3	9.3
	p value versus baseline	0.97	0.53
	p value versus placebo	0.58	

**ALT (U/L)**			

Baseline	mean	31.2	44.8
Change from baseline	LS mean	0.1	−5.1
	SE of LS mean	1.2	2.2
	p value versus baseline	0.93	0.037
	p value versus placebo	0.062	

**AST (U/L)**			

Baseline	mean	25.1	27.8
Change from baseline	LS mean	−0.3	−1.2
	SE of LS mean	1.1	1.9
	p value versus baseline	0.81	0.53
	p value versus placebo	0.67	

**GGT (U/L)**			

Baseline	mean	34.3	58.8
Change from baseline	LS mean	−10.2	1.0
	SE of LS mean	2.1	3.8
	p value versus baseline	0.0005	0.80
	p value versus placebo	0.029	

**hsCRP (mg/L)**		**(n = 11)**	

	mean	7.7	3.4
	LS mean	−1.0	0.5
	SE of LS mean	1.4	2.5
	p value versus baseline	0.50	0.85
	p value versus placebo	0.64	

**MCP-1 (pg/mL)**			

	mean	300.4	228.0
	LS mean	7.9	−31.9
	SE of LS mean	12.7	22.8
	p value versus baseline	0.55	0.19
	p value versus placebo	0.16	

The indicated test results were obtained at baseline and following four weeks of treatment with PXL770. Values for 4 placebo subjects and 12 PXL770 treated subjects are shown except where noted (∗n = 10-11 due to incomplete availability of samples from the OGTT). The total AUC or incremental AUC’s (iAUC) for glucose, C-peptide, insulin, and free fatty acids (FFA) were calculated based on results obtained during oral glucose tolerance tests (OGTT). Indices of insulin sensitivity (HOMA-IR, Matsuda, OGIS) were calculated as described in StarMethods. Other results were based on baseline or pre-dose samples obtained under fasting conditions (hsCRP, highly sensitive C-reactive protein; MCP-1, monocyte chemoattractant protein; TG, total triglycerides; ALT, alanine aminotransferase; AST, aspartate aminotransferase; GGT, γ−glutamyl transferase).

Importantly, there was no evidence of changes in body weight (Table S2); this suggests that the potential changes in glycemia (OGTT) and insulin sensitivity indices are not attributable to weight loss. There were also no changes in ALT or AST levels in treated subjects (Table 1), where mean baseline values were close to the upper limit of normal reference ranges: ALT £ 33 IU/L for females and £ 41 for males, AST £ 32 for females and £ 40 for males (Table 1); however, a reduction in γ−glutamyl transferase (GGT) versus baseline (and versus placebo) was observed (Table 1). In addition, there were no significant effects on fasting total triglycerides (TG), LDL and HDL cholesterol values, or apolipoprotein B levels (Table 1). Finally, no significant changes in adiponectin levels or selected biomarkers of inflammation, C-reactive protein (hsCRP) and monocyte chemoattractant protein-1 (MCP-1), were observed (Table 1); mean baseline levels for these inflammation markers were also not elevated at baseline.

Given the appearance of substantial variability in the DNL response to PXL770 treatment (Figure 3B), we also conducted a *post hoc* analysis of DNL results in an attempt to assess potential correlations with better responsiveness. Subjects treated with PXL770 were readily separated into two dichotomous and equally sized groups based on peak DNL suppression of greater or lesser than 20% (Table 2). Importantly, no significant differences in PK (C_max_ and AUC) were apparent to explain the differences in DNL response between these two groups. Of interest, better DNL responders had statistically greater degrees of glucose intolerance and insulin resistance at baseline along with trends toward higher plasma fasting glucose, insulin, and TG levels (Table 2). These findings are potentially consistent with available literature showing that tissue levels of endogenous AMPK activity are suppressed by greater degrees of metabolic dysfunction.^29^

**Table 2:** Analysis of *De Novo* lipogenesis responders versus low responders

	*Responders (N = 6)*			*Low responders (N = 6)*			
	Mean	SE.	Median	Mean	SE.	Median	p value
**Peak DNL Inhibition, %**	**−37**	**3.2**	**−38**	**−3**	**6.9**	**−8**	**0.0039**^c^

Female n (%)	5 (83.3%)			3 (50%)			0.221
Age, years	44.2	5.9	44.5	53.4	4.7	51.5	0.3367
BMI, kg/m^2^	39.3	2.7	39.0	37.6	3.3	36.0	0.6310
FPG, mg/dL^d^	133.2^a^	19.6	123	96.8	4.0	96	0.0782^d^
Insulin, μU/mL^d^	68.2	21.4	57.6	28.8	5.3	23.2	0.0782^d^
AUC glucose, mg∗h/dL^c^	624	87	566	424	19	416	0.0065^c^
Matsuda index^c^	0.55	0.08	0.48	1.41	0.18	1.33	0.0090^c^
OGIS index^c^	174.3	17.3	183.3	274.7	37.2	254.7	0.0283^c^
AdipoIR, mEq/L × pmol/L	213	74	177	83	16	64	0.1093
Adiponectin, ug/mL	3.1	0.5	2.9	4.5	0.5	4.7	0.1488
FFA, mmol/L	0.43	0.05	0.44	0.42	0.02	0.43	1.0000
TG, mg/dL^d^	179.8	24.0	176.5	122.5	20.1	102	0.0547^d^
ALT, U/L	34.5	7.6	37	28	2.5	27	0.4217
PXL770 plasma Cmax^b^, μg/mL	25.8	2.6	26.7	21.3	5.0	17.1	NS
PXL770 plasma AUC_0-12 h_^b^, μg.h/mL	124.9	7.8	123.0	94.7	15.6	71.4	NS

Post-hoc analysis of PXL770 treated *de novo* lipogenesis (DNL) responders versus low responders. Subjects who achieved^3^ 20% suppression of peak fructose-stimulated DNL at day 28 versus baseline (day 1) were considered to be responders. Comparison of mean and median (±SE) baseline values for the indicated parameters from responder versus low responder groups is reported. AUC glucose is based on results from oral glucose tolerance tests.

a One subject was found to have fasting glucose in the diabetic range at randomization.

b PK parameters were assessed on day 28 during the DNL assessment.

c Statistically significant differences (p < 0.05).

d Additional trends of potential interest are italicized.

### Additional plasma lipid assessments

**Figure 4:**
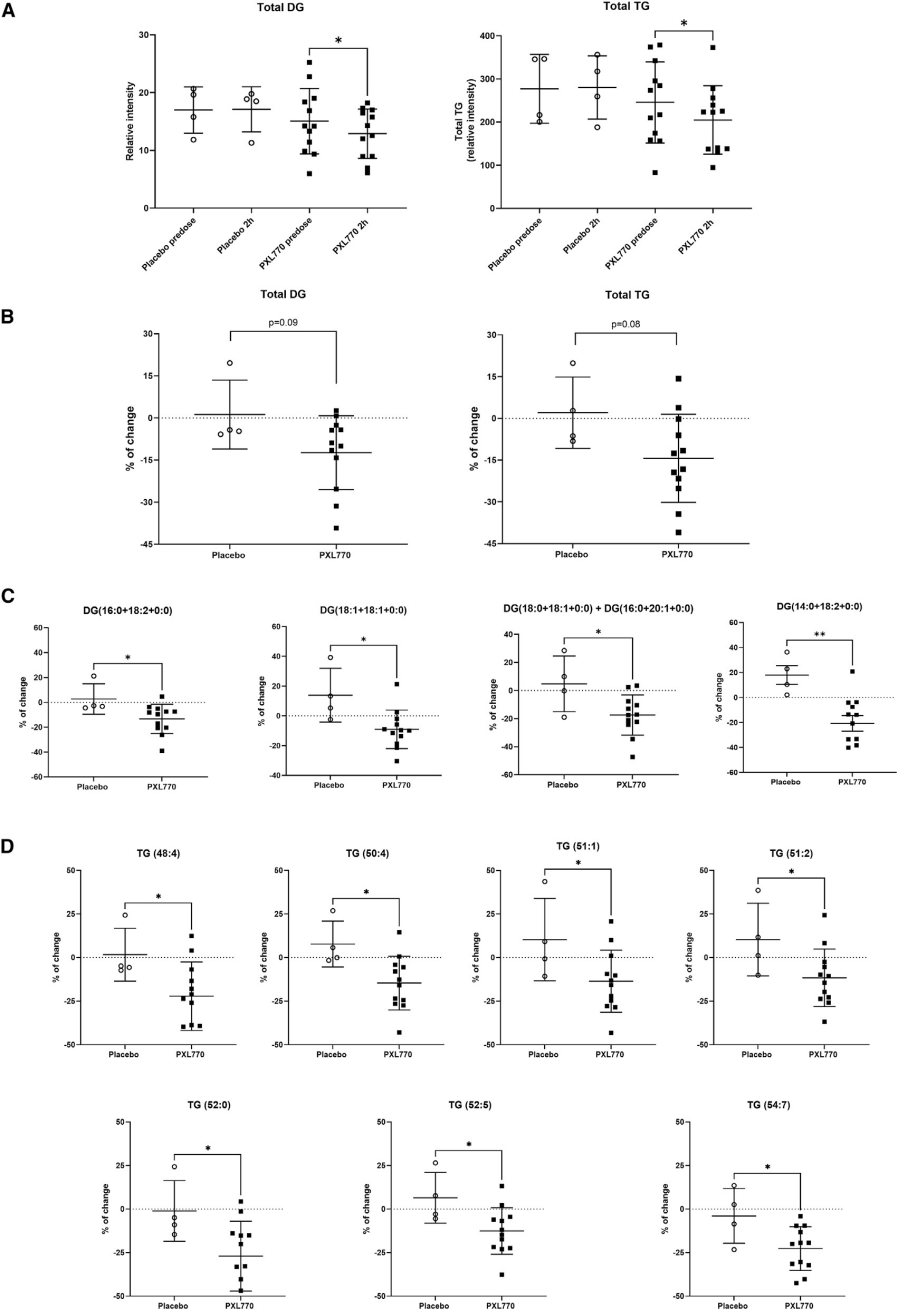
PXL770 acutely decreases diacylglycerides and triacylglycerols Plasma samples were assayed using liquid chromatography coupled to mass spectrometry to detect and quantitate a range of selected circulating lipids. Day 14 samples were collected from both placebo and PXL770 treated subjects at pre-dose (trough) and 2 h post-dose (Cmax) time points; both under fasted conditions. Plasma total DG and TG are expressed as relative intensity (sum of the normalized peak areas of all detected DG and TG species) pre-dose and 2 h post-dose at day 14 for placebo (n = 4 patients) and PXL770 (n = 12 patients); mean ± SD, ∗p < 0.05 versus day 14 pre-dose (A). Change from pre-dose to 2 h post-dose on day 14 for total (B) and specific DG (C) and TG (D); mean ± SD, ∗p < 0.05 versus placebo.

Although standard laboratory measurements of fasting lipids were not significantly affected by exposure to PXL770, we pursued a broader investigation of selected lipid classes including circulating diacylglycerols (DG), triacylglycerols (TG), ceramides, and sphingomyelins. For this purpose, liquid chromatography and mass spectrometry was used to analyze plasma samples that were obtained from study subjects at three time points: baseline (fasting day −2); pre-dose on day 14 (fasting); and 2 h post-dose on day 14 (fasting). This latter time point coincides with C_max_ for PXL770 exposure. Overall, there were no changes in selected lipids when baseline samples were compared to pre-dose day 14 samples. In contrast, a significant decrease in total DG and TG (−14.4%, p = 0.01, and −16.6%, p = 0.01, respectively) was evident when an assessment of samples from day 14 pre- versus post-dose was made (Figure 4A). Changes from day 14 pre- versus post-dose were then compared between PXL770- and placebo-treated subjects. Although this reduction in total DG and TG induced by PXL770 dosing did not reach significance when compared to placebo (p = 0.09 and p = 0.08, respectively), significant differences in several individual DG and TG species versus placebo were observed; specifically 4 DGs of 15 analyzed and 19 TGs of 88 analyzed achieved significance (Figures 4B, 4C, and 4D and Table S2). PXL770 treatment did not appear to change post-dose total ceramides and sphingomyelins (versus pre-dose or versus placebo; Table S2).

### Safety

All adverse events (AE) reported upon initiation of PXL770 or placebo were mild in severity, with the exception of one moderate AE (diarrhea in the PXL770 group). No serious AE, death, or severe AE occurred. The most commonly observed AE were events of diarrhea reported by 6 patients (46%) in the PXL770 group and 4 patients (100%) in the placebo group and occurring mainly on the days of DNL testing with a similar occurrence at baseline and at the end of treatment (Table 3). This is consistent with the administration of high and repeated amounts of fructose.^30^ Since AMPK activation has the potential to reduce protein synthesis,^1^ it may be relevant to note that serum albumin and total protein concentrations were not meaningfully affected (Table S4). In addition, no clinical pathology effects attributed to reduced hepatic protein synthesis were evident in chronic preclinical toxicology studies (data not shown).

**Table 3:** Adverse events

	PXL770 500 mg (QD)	Placebo (QD)
Number of patients	13	4
Any TEAE	7 (53.8%)	4 (100%)
Any related TEAE	2 (15.4%)	1 (25.0%)
Any SAE	0	0
TEAE leading to discontinuation	0	0
TEAE leading to death	0	0
Any TEAEs		
Diarrhea	6 (46.2%)	4 (100%)
Nausea	1 (7.7%)	0
Dizziness	1 (7.7%)	0
Headache	1 (7.7%)	0
Thrombophebitis superficial	0	1 (25.0%)
Any related TEAEs		
Diarrhea^a^	1 (7.7%)	1 (25.0%)
Dizziness	1 (7.7%)	0

a Most episodes of diarrhea were coincident with the administration of oral fructose during the DNL test as discussed in Results.

## Discussion

In the present study, we endeavored to assess the effects of direct AMPK activation in humans with features of the metabolic syndrome: specifically, in subjects with insulin resistance and NAFLD with increased BMI. For this purpose, we used PXL770, a well-characterized orally bioavailable thienopyridone small molecule that functions as a potent and selective allosteric activator of multiple recombinant human AMPK complexes. PXL770 has also been shown to activate AMPK in several rodent and human-derived cell types and to ameliorate core aspects of disease in animal models of T2D and NASH.^23^

The primary endpoint of the study was to examine the PK profile in this patient population; other endpoints were considered exploratory. Steady state PK measurements revealed that PXL770 was well absorbed and displayed a long terminal half-life that supports once-daily oral dosing. The 500 mg daily dose was chosen based on prior phase 1 studies in healthy volunteers where this dose was well tolerated and exposure levels had reached a plateau; in addition, appropriate preclinical toxicology results supported this dosing level and the treatment duration of in the present trial (data not shown). The plasma concentration-time curve in NAFLD patients was also similar to the profile observed in prior Phase 1 trials. A negative food effect was evident when comparing fed versus fasted PK parameters after PXL770 administration as capsule formulation (Figure 2). Mean plasma exposure in the fed state observed in this study was still within the range associated with efficacy in rodent efficacy studies (data not shown).

PD measurements revealed effects that are indicative of target engagement and were potentially suggestive of the potential for longer term efficacy in metabolic disease. Fructose-stimulated DNL was significantly suppressed by PXL770. This represents a classical effect of AMPK activation as ACC is a direct substrate for the enzyme where phosphorylation results in inhibition leading to reduced lipogenesis.^31^ Importantly, both pharmacologic and genetic AMPK activation have been consistently reported to reduce DNL and hepatic steatosis in preclinical models;^32^, ^33^, ^34^, ^35^, ^36^ in previous experiments, we have also demonstrated that PXL770 reduces steatosis in rodents and inhibits DNL with equal potency in isolated human versus mouse hepatocytes. The reduction in DNL may also be predictive of the potential for longer term decreases in liver steatosis because NAFLD and NASH are characterized by substantial increases in DNL.^17^^,^^37^ It would also be of interest to assess fractional DNL in response to typical meal intake rather than only in response to fructose. Increased FFA flux from adipose tissue also represents an additional major contributor to hepatic lipid deposition;^18^ however, the extent to which AMPK can directly inhibit lipolysis is uncertain.^4^ Although we did not detect decreases in FFA concentrations in response to PXL770, we cannot exclude a potential effect on lipolysis, because absolute FFA levels were normal and lipolysis was not directly measured.

Given variability seen in the DNL response to PXL770 (Figure 3), we were motivated to assess potential differences in the phenotype of subjects with greater versus lesser evidence of target engagement. This comparison revealed that “responder” subjects were more glucose intolerant and insulin resistant and tended to also have higher fasting glucose and TG levels at baseline. These findings are consistent with prior literature showing that greater degrees of metabolic dysfunction (or inflammation) are associated with lower levels of endogenous AMPK activity.^29^ For example, high glucose suppresses AMPK activity in human cells and *in vivo* in rat tissues.^38^, ^39^, ^40^ Moreover, adipose tissue AMPK activity is reduced in obese insulin-resistant humans^41^^,^^42^ and exercise-stimulated skeletal muscle AMPK activity is attenuated in obese patients with T2D.^43^ Although it was not feasible to measure AMPK activity in tissues from subjects enrolled in our study, we speculate that greater responses to pharmacologic AMPK activation might be predicted to occur in individuals with lower endogeous AMPK “tone.” Future studies will help confirm this hypothesis.

Potential improvements in FPG and glucose levels during the OGTT were noted in subjects treated with PXL770 relative to baseline measurements, although these effects were not significant when compared to placebo. Several indices of insulin sensitivity appeared to be improved in PXL770-treated subjects. However, it should be noted that there was a substantial imbalance between mean baseline values of HOMA-IR in the PXL770 group versus placebo (Table S1). As HOMA-IR is based on fasting parameters, this difference suggests a greater degree of hepatic insulin resistance in the PXL770 group; in contrast, no obvious baseline differences in other indices (Matsuda and OGIS), which are more likely to be measures of peripheral insulin sensitivity, were noted. Improved insulin sensitivity in response to PXL770 treatment may be consistent with predictions based on several lines of preclinical evidence and suggest the potential for longer term therapeutic utility in diabetes as well as in the context of NAFLD-NASH^15^^,^^16^ or other disorders such as polycystic ovary syndrome^44^ in which insulin resistance is a central component of pathophysiology. Prior studies with a variety of pharmacologic AMPK activators, both direct and indirect, have yielded consistent effects to ameliorate hyperglycemia in both rodent^45^, ^46^, ^47^ and non-human primate models.^47^ Effects in other preclinical model systems have suggested the potential for AMPK activation to enhance insulin sensitivity; this includes the effects of AMPK to enhance insulin signaling^6^ and GLUT4 glucose transporter-mediated glucose uptake;^48^ the latter effect of AMPK has also been implicated as a major mechanism for increased muscle glucose uptake and metabolism during exercise.^10^ Of note, PXL770 was recently shown to produce substantial improvements in whole body insulin sensitivity (via hyperinsulinemic clamp) in high-fat-diet-induced obese insulin-resistant mice.^23^ Further studies involving clamp experiments will be required to confirm that direct AMPK activation has a bona fide effect on insulin sensitivity in humans.

Variable effects of AMPK activation on circulating total cholesterol and TG have been reported in studies of direct AMPK activators in animal models.^10^^,^^49^ In the current study, no effects of PXL770 were noted when fasting lipids were assessed (in association with low trough drug levels). However, when selected lipid classes were examined using samples obtained at or near the time of Cmax drug exposure, a potential acute effect to lower certain DG and TG species was evident. These findings may be important given that elevated plasma levels of these classes are associated with NAFLD-NASH^50^, ^51^, ^52^ and have been suggested to be potential biomarkers of steatosis severity and disease progression.^50^^,^^53^ Several of the DG and TG species affected by PXL770 (DG 32:2, DG 34:2, DG 36:1, DG 36:2, TG 50:4, and TG 48:4) have also been associated with increased lobular inflammation, ballooning, steatosis, insulin resistance, HbA1c, and visceral adipose tissue.^51^ Changes in DG are of potentially broad importance given that this lipid class has been implicated as “lipotoxic” in the pathophysiology of a range of metabolic diseases.^54^^,^^55^

We did not detect significant effects on several parameters that have been previously suggested as linked to AMPK activation.^13^ This includes a lack of effect to reduce markers of inflammation: hsCRP or MCP-1. ALT and AST levels were also unaffected although GGT levels were reduced; this suggests the potential for a liver benefit given that GGT levels are frequently elevated (and may confer a higher risk of progression) in the context of NAFLD.^56^ It should be noted that the aforementioned parameters were generally within normal reference ranges at baseline.

To our knowledge, no other direct AMPK activator has been previously studied in human patients with metabolic disease. Single doses of one such molecule^49^ (PF-06409577) were administered to healthy subjects (ClinicalTrials.gov identifier NCT02286882); however, no results have been reported to date. Attempts to discover and develop direct allosteric AMPK activators have also been hindered by multiple challenges, including very low oral bioavailability as seen with A-769662^45^ or the finding of untoward safety findings in preclinical species as reported with MK-8722.^47^

Steneberg et al. reported effects of a molecule referred to as O304 in both diet-induced obese mice and in a small number of patients with T2D.^57^ Importantly, O304 does not directly activate AMPK; it appears to augment AMPK activation exclusively via a mechanism involving inhibition of dephosphorylation; thus, its effects *in vivo* might reflect actions that are also AMPK independent. Several commonly prescribed drugs, most notably metformin but also thiazolidinediones such as pioglitazone, have been reported to enhance AMPK activity through indirect (upstream) pathways.^58^^,^^59^ Importantly, these examples suggest that prolonged AMPK activation, at least at low levels, is likely to be safe. However, these therapies also clearly operate through additional non-AMPK related pathways, including the inhibition of mitochondrial glycerophosphate dehydrogenase^60^ and PPARγ activation plus unrelated non-genomic pathways,^61^^,^^62^ respectively.

Safety was carefully monitored in this study. No individual serious adverse events were attributed to PXL770 and there was no evidence of any safety signals that exceeded effects seen in the placebo group nor any evidence of laboratory or ECG-related abnormalities of concern. This safety and tolerability profile was also consonant with results obtained in phase I with healthy subjects (data not shown).

AMPK activation has been proposed as a therapeutic target for a broad range of diseases.^10^ The experimental medicine study described herein demonstrates that this approach, via leveraging a small molecule allosteric activator, could achieve systemic measurable levels of target engagement. Effects on selected metabolic efficacy signals suggest the potential for translation from animals to humans. However, in light of the limitations of this study, noted below, effects on these exploratory pharmacodynamic endpoints should be interpreted with caution. Our findings also indicate that AMPK activation is well tolerated and associated with adequate safety to allow for longer term future studies.

### Limitations of the study

This was a Phase 1B study with a limited number of subjects where the primary outcome was to determine PK parameters of PXL770. Other measured parameters were exploratory. The inclusion of a very small (n = 4) number of placebo-treated subjects did not allow for an appropriate control group with which to accurately compare potential effects of PXL770 on pharmacodynamic parameters; there was also a potential study effect on selected parameters (e.g., FPG and glucose AUC) evidenced by potential changes in the placebo group. In addition, statistical analyses of exploratory endpoints did not include adjustments for multiplicity and should therefore be interpreted with caution. Plasma exposure levels were also substantially affected by food intake; this is likely to have significantly limited the extent of potential effects on efficacy-related parameters such as fasting glucose and lipid values. Efforts to address this issue in order to optimize plasma exposure levels of PXL770 should be considered to potentially allow for better exposure and assessment of the effects of direct AMPK activation in humans with this molecule. The short time frame (28 days) of this study was also a limitation and may have precluded the ability to detect additional metabolic efficacy-related signals such as weight loss and decreases in lipids or inflammation parameters.

## STAR★Methods

### Key resources table

**Table:** 

REAGENT or RESOURCE	SOURCE	IDENTIFIER
**Biological samples**		

Subjects recruitment and sample collection	High Point Clinical Trials Center https://highpointctc.com/	NA
	Texas Liver Institute https://txliver.com/	

**Chemicals, peptides, and recombinant proteins**		

Deuterated water (D2O, 70% ± 5 %)	Cambridge Isotope Laboratories https://www.isotope.com/	NA
Fructose	Archer Daniels Midland https://www.adm.com/	Product Code: 010034
PXL770	Poxel SA https://www.poxelpharma.com	NA

**Critical commercial assays**		

Biochemistry/Safety analysis including glucose, insulin, C-peptide, lipids, inflammatory biomarker and liver enzymes	Cerba Research https://www.cerbaresearch.com/	NA
Lipidomic analysis	One Way Liver, S.L https://www.owlmetabolomics.com/liver-disease-diagnosis.aspx	NA
Pharmacokinetic samples analysis	Charles River Laboratories https://www.criver.com/	NA
*De novo* lipogenesis	Metabolic Solutions https://www.metsol.com/	NA

**Software and algorithms**		

Phoenix® WinNonlin®	Certara https://www.certara.com	Version 8.1
TargetLynx application manager/MassLynx	Waters Corp https://www.waters.com	Version 4.1
SAS	SAS Institute https://www.sas.com	Version 9.4

### Resource availability

#### Lead contact

Further information and requests for resources and reagents should be directed to the Lead Contact, Pierre Theurey (pierre.theurey@poxelpharma.com). Limited quantities of PXL770 may be available under a Material Transfer Agreement.

#### Materials availability

All unique/stable reagents generated in this study are available from the lead contact with a completed Materials Transfer Agreement.

### Experimental model and subject details

This was an exploratory Phase 1b, double-blind, placebo-controlled, randomized 4-week study conducted at 2 sites in USA. After screening, eligible subjects were randomized in a 3:1 ratio (PXL770: placebo) to receive either PXL770 500 mg QD or matched placebo orally. Subjects were given 2 hard gelatin capsules QD (2 x PXL770, 250 mg QD or placebo). The placebo capsules were identical in size and appearance to the active drug capsules.

Adults (18-75 years) with NAFLD based on a CAP score > 300 dB/m by transient elastography (Fibroscan™) as a validated predictor of hepatic steatosis^24^, and HOMA-IR > 2.5^25^ but without evidence of diabetes (fasting plasma glucose < 126 mg/dL) and elevated ALT concentrations (> 20 U/l in women and > 30 U/L in men) were enrolled. This ALT eligibility criterion was secondarily removed following a protocol amendment on Nov 07, 2019 when it was deemed to be too restrictive.

Patients were excluded if they: had evidence of advanced fibrosis as assessed by liver stiffness measurement (Fibroscan) ^3^ 14 kPa, evidence of hepatic impairment, AST/ALT > 200 U/L, clinically significant acute or chronic liver disease unrelated to non-alcoholic steatohepatitis; any cardiovascular event or evidence of active cardiovascular disease within 6 months of screening.

### Method details

#### Study design

The study protocol was approved by local ethics committees before trial initiation. The study was conducted in compliance with International Conference on Harmonization, E6 Good Clinical Practice (GCP) guidelines. Written informed consent was obtained from all patients before beginning any study-related activities. Patients were allocated to treatment groups via an interactive web response system. Investigators, staff, patients, and the funder remained masked throughout the study period. After a screening period, study treatment was administered for 4 weeks. Subjects were then followed up for 1 additional week. Subjects were confined at the study site for single day periods of time to accommodate testing that included PK measurements, OGTT tests and fructose-stimulated DNL measurements (Figure 1A).

#### Pharmacokinetics

Blood samples for PK analysis of PXL770 were collected from each subject predose on Days 14 (fasting conditions) and 26 (fed conditions – standardized breakfast) at 30 min, 45 min, and 1, 1.5, 2, 2.5, 3, 4, 6, 8, 12, 16, and 24 h postdose, and on Day 28 during the DNL assessment at the same time points as employed in the DNL procedure. Plasma concentrations of PXL770 were analyzed using validated protein precipitation and high-performance liquid chromatography tandem mass spectroscopy (HPLC-MS/MS) methods. Pharmacokinetic samples collected from subjects who received placebo were not analyzed for PXL770. The analytical phase was carried out in accordance with GCP guidelines. The lower limit of quantification for PXL770 plasma concentrations was 10 ng/mL. Pharmacokinetic data were analyzed using validated PK analysis software (Phoenix® WinNonlin® version 8.1, Certara, Princeton, NJ). All PK analyses were performed, saved, and audit trailed in a 21 Code of Federal Regulations Part 11 compliant Oracle database (Phoenix® Knowledgebase Server Online). All plasma PK parameters were calculated using actual time points relative to the time of study drug administration. If actual time was missing, nominal time was used.

#### *De novo* lipogenesis (DNL)

Fractional hepatic DNL was assessed at baseline and following the administration of PXL770 or placebo for 4 weeks. The DNL assessment was performed after a period of 48 h of restricted diet and physical activity. A fructose-loading procedure was employed to induce a DNL signal, using D_2_O as a tracer to measure the fractional contribution of hepatic DNL to triglyceride-palmitate^63^. The night prior to the DNL procedure, subjects consumed 200 mL of 70% D_2_O divided into 4 doses, each separated by a minimum of 1.5 h, under the supervision of the clinical study site staff. Other than D_2_O administration, subjects were fasted overnight. Following the overnight fast, subjects then received small volumes of a chilled oral fructose solution (50-75mL, 0.25 g/kg body weight) every 30 min for a total of 18 doses. Blood samples were collected every h for the assessment of fructose stimulated DNL for up to 10 h. Body water enrichment was measured from blood samples collected at select time points during the D_2_O labeling periods and during the fructose feeding. Fructose-induced fractional DNL was determined by calculating the change from fasting baseline DNL at each post-dose time point by subtracting individual baseline values from time-matched active values for each subject. In addition, fractional DNL area under the effect curve was determined. Peak DNL, as defined by the average of the 3 last time points (8, 9 and 10 h), was also assessed.

#### Oral glucose tolerance testing (OGTT)

OGTT tests were conducted at baseline and following the administration of PXL770 or placebo for 4 weeks. The OGTT was performed after an overnight fast and after at last 48 h of an unrestricted diet and physical activity. Subjects received their PXL770 or placebo capsule 2 h before ingesting 75 g of anhydrous glucose dissolved in 200 mL of water. Blood samples were collected for glucose, insulin, C-peptide and FFA measurements at 30, 60, 90, 120, 150 and 180 min. The total and incremental AUC for glucose was calculated using the trapezoidal rule for the evaluation of the total blood glucose response versus time during OGTT. OGTT derived insulin sensitivity indices were also calculated as previously described: HOMA-IR^25^, Matsuda index,^27^ and OGIS^28^.

#### Additional pharmacodynamic measurements

Additional secondary efficacy parameters including lipids, inflammatory biomarker and liver enzymes were analyzed at baseline and after 4 weeks of treatment.

#### Additional lipid assessments

Plasma samples were collected under fasted conditions: at baseline, day 14 pre-dose and day 14, 2 h post dose in NAFLD patients. Samples were immediately frozen and later analyzed by ultraperformance liquid chromatography coupled to mass spectrometry (UHPLC-MS). The analysis focused on determination of glycerolipids (15 different diacylglycerols and 94 different triacylglycerols). Briefly, proteins were precipitated by adding chloroform:methanol (2:1) containing internal standards. Homogenization of the resulting mixture was performed using a Precellys 24 homogenizer (Bertin Technologies, Montigny-le-Bretonneux, France) at 6500 rpm for 45 s x 1 round. Homogenized samples were incubated at −20°C for 1 h and after vortexing, a 500 μL aliquot was collected. The supernatants were mixed with ammonium hydroxide in H_2_O (pH 9) and incubated for 1 h at −20°C. After centrifugation, the organic phase was collected and dried under vacuum. Dried extracts were reconstituted in acetonitrile / isopropanol (1:1) for LC-MS analysis using standard methods^64^. All data were processed using the TargetLynx application manager for MassLynx 4.1 software (Waters Corp., Milford, USA). Intra- and inter-batch normalization was performed by inclusion of multiple internal standards for each metabolite and pool calibration response correction, as previously described ^65^.

#### Safety assessments

Safety was evaluated by assessment of clinical laboratory tests (hematology, coagulation, chemistry, urinalysis, virus serology, urine drug screen and pregnancy test), physical examinations, vital sign measurements (1 heart rate measurement and 3 blood pressure measurements after at least 5 min of rest), ECG and documentation of adverse events. Laboratory tests were performed at day −3, day 14 and end of study visit. Vital signs were performed at screening, days −3, −2; −1, 1, 14, 27, 28 and at end of study visit. Triplicate 12-lead ECG (conducted 5 min apart) were performed pre-dose and 2 h post dose on day, day 14 and at the end of study visit. All safety data were collected from the time of the informed consent signature to the end of the study.

### Quantification and statistical analysis

Statistical analyses were performed using SAS 9.4. Continuous variables were summarized using descriptive statistics and categorical variables were summarized using counts and percentages. Efficacy endpoints were all exploratory and therefore no adjustment for multiplicity was made. The mean change from baseline within group were assessed with paired t tests and 95% confidence intervals. Two populations were used for statistical analyses of efficacy endpoints: the modified ITT (mITT) including all subjects with a baseline value and at least one post-baseline efficacy assessment; the Per Protocol Set (PPS) including all subjects who completed at least 90% of the 4-week treatment regimen, had overall compliance ^3^ 90%, had at least 1 measured study drug concentration and who did not have any major protocol deviations. Unless otherwise noted, all data and corresponding statistics were based on mITT.

For lipidomic measurements obtained using UHPLC-MS, no specific hypothesis was considered; post hoc analyses were performed on bio-banked samples as an exploratory assessment. Data are expressed as mean ± SD. Both individual lipid species and relative abundances of all detected species within a given class (e.g., DG) were determined; in the latter case, as the sum of all normalized peak areas which was expressed in relative intensity units. Differences in total DG or total TG between D14 2 h post-dose versus D14 pre-dose were determined using paired Student’s t test. The differences between placebo and PXL770 group were assessed using unpaired Student’s -test. A p value of < 0.05 (versus placebo) was chosen to represent significance.

### Additional resources

Study was registered in ClinicalTrials.gov as NCT03950882.

## Data Availability

The clinical data reported in this study cannot be deposited in a public repository because of privacy and ethical reasons. To request access, contact the lead contact at pierre.theurey@poxelpharma.com. This paper does not report original code. Any additional information required to reanalyze the data reported in this paper is available from the lead contact upon request.

## Supplemental information

Document S1. Tables S1–S4

Document S2. Article plus supplemental information
