# Supplementary material for: Pharmacodynamic effects of direct AMP kinase activation in humans with insulin resistance and non-alcoholic fatty liver disease: A phase 1b study
Source: Cell Rep Med. 2021 Dec 21;2(12):100474. doi: 10.1016/j.xcrm.2021.100474 (PMC8714938; doi:10.1016/j.xcrm.2021.100474)
Supplement: Document S1. Tables S1–S4 [file mmc1.pdf]

Cell Reports Medicine, Volume *2*

Supplemental information

Pharmacodynamic effects of direct AMP kinase activation in humans with insulin resistance and non-alcoholic fatty liver disease: A phase 1b study

Pascale Fouqueray, Sebastien Bolze, Julie Dubourg, Sophie Hallakou-Bozec, Pierre Theurey, Jean-Marie Grouin, Clémence Chevalier, Pascale Gluais-Dagorn, David E. Moller, and Kenneth Cusi

	PXL770 500 mg (QD)	Placebo (QD)
Number of patients	13	4
Age, years	48.5 (12.8)	44.3 (16.9)
Gender		
Female (%)	9 (69.2%)	2 (50.0%)
Male (%)	5 (30.8%)	2 (50.0%)
Weight, kg	109.2 (25.99)	108.3 (36.29)
BMI, kg/m^2	38.1 (6.9)	36.4 (5.1)
CAP, dB/m	360.4 (33.5)	317.5 (6.0)
FPG, mg/dL	115.2 (36.5)	94.5 (10.6)
HOMA-IR	14.7 (15.2)	8.8 (3.3)

Supplemental Table I. Baseline Clinical Characteristics of Study Subjects. Data are mean (SD) or n (%) unless otherwise specified. BMI: body mass index; CAP: Controlled attenuation parameter; QD: once daily; FPG: Fasting Plasma Glucose; HOMA-IR: Homeostatic Model Assessment of Insulin Resistance. Related to Figure 1.

	Placebo		PXL770	
	Baseline (n=4)	Week 5 (EOS, n=4)	Baseline (n=13)	Week 5 (EOS, n=12)
**Body weight (kg) – Mean (SD)**	108.300 (36.294)	109.600 (36.364)	109.208 (25.995)	110.883 (25.612)

Supplemental Table II. Body Weight (kg) at Baseline and End of Study (EOS) – Week 5. Related to Table 2.

% change post-dose (2H) vs pre-dose		Placebo		PXL770		
		Mean	SD	Mean	SD	T test vs placebo
TG(42:0)	*TG(14:0+12:0+16:0)*	-11,415	32,467	-27,172	32,746	0,416
TG(44:0)	*TG(16:0+16:0+12:0) + TG(16:0+14:0+14:0)*	-6,231	15,647	-23,866	19,879	0,147
TG(44:1)	*TG(14:0+18:1+12:0) + TG(16:0+16:1+12:0)*	-5,944	22,823	-25,463	29,492	0,268
TG(44:2)	*TG(14:0+18:2+12:0)*	-20,262	24,679	-29,291	28,4	0,546
TG(45:0)	*TG(16:0+15:0+14:0)*	-4,82	8,536	-11,081	23,65	0,615
TG(46:0)	*TG(16:0+16:0+14:0)*	-4,682	11,21	-20,824	15,955	0,106
TG(46:1)	*TG(12:0+18:1+16:0)*	1,415	9,608	-17,96	17,567	0,065
TG(36:0)	*TG(14:0+10:0+12:0)*	16,217	21,14	-4,932	18,365	0,079
TG(46:2)	*TG(14:0+18:2+14:0) + TG(16:1+18:1+12:0)*	0,638	16,765	-20,576	23,145	0,119
TG(46:3)	*TG(18:2+16:1+12:0 + TG(18:1+16:2+12:0) + TG(10:0+18:2+18:1)*	-4,839	20,835	-18,797	28,685	0,388
TG(40:0)	*TG(12:0+16:0+12:0) + TG(10:0+16:0+14:0)*	-14,555	23,479	-27,709	29,572	0,416
TG(47:0)	*TG(16:0+16:0+15:0)*	5,301	16,156	-16,275	20,566	0,086
TG(42:2)	*TG(12:0+18:2+12:0) + TG(10:0+18:2+14:0)*	8,814	68,897	-35,704	35,937	0,1
TG(43:0)	*TG(12:0+15:0+16:0) + TG(10:0+16:0+17:0) + TG(12:0+14:0+17:0)*	-4,336	7,258	-6,226	43,668	0,917
TG(43:1)	*TG(10:0+18:1+15:0) + TG(12:0+17:1+14:0)*	-8,102	53,604	16,704	95,158	0,696
TG(44:3)	*TG(8:0+18:1+18:2) + TG(10:0+16:1+18:2) + TG(10:0+18:3+16:0) + TG(10:0+20:3+14:0)*	-7,719	49,854	-22,445	37,785	0,557
TG(46:3)	*TG(12:0+18:3+16:0)*	-5,512	21,235	-21,191	26,768	0,314
TG(47:1)	*TG(18:1+14:0+15:0) + TG(16:1+17:0+14:0)*	10,481	18,521	-10,185	28,097	0,203
TG(47:2)	*TG(12:0+18:2+17:0) + TG(12:0+18:1+17:1) + TG(14:0+18:2+15:0)*	3,905	12,576	-5,159	44,114	0,686
TG(48:0)	*TG(16:0/16:0/16:0)*	-1,589	15,295	-17,959	14,633	0,089
TG(52:4)	*TG(16:0+20:4+16:0)*	-4,216	21,049	-15,298	18,216	0,326
TG(52:6)	*TG(16:1+18:3+18:2)*	4,979	13,355	-14,688	16,325	0,049
TG(53:5)	*TG(17:1+18:2+18:2)*	10,078	18,987	-12,613	18,086	0,048
TG(48:1)	*TG(14:0+18:1+16:0) + TG(16:0+16:1+16:0)*	1,399	11,476	-15,687	14,044	0,058
TG(55:1)	*TG(16:0+21:0+18:1) + TG(18:0+18:1+19:0)*	-17,881	19,959	-20,917	48,836	0,946
TG(55:2)	*TG(18:1+18:1+19:0) + TG(18:0+18:1+19:1)*	6,341	25,162	-16,867	22,585	0,108
TG(55:3)	*TG(18:1+18:1+19:1) + TG(18:2+19:0+18:1)*	8,951	15,61	-13,049	16,879	0,039
TG(55:4)	*TG(18:1+19:1+18:2)*	16,732	15,872	-9,348	19,715	0,031
TG(58:5)	*TG(18:0+22:4+18:1)*	7,616	14,425	-4,63	20,211	0,245
TG(48:2)	*TG(14:0+18:1+16:1) + TG(16:0+18:2+14:0)*	2,173	13,853	-15,222	14,905	0,071
TG(48:3)	*TG(18:1+18:2+12:0)*	5,027	14,342	-17,361	19,198	0,055
TG(48:4)	*TG(18:2+18:2+12:0) + TG(18:1+18:3+12:0)*	1,635	15,15	-22,256	19,643	0,045
TG(49:0)	*TG(16:0+17:0+16:0) + TG(18:0+17:0+14:0)*	4,762	19,803	-16,839	19,367	0,08
TG(49:1)	*TG(15:0+18:1+16:0)*	8,245	16,193	-13,214	18,449	0,062
TG(49:2)	*TG(15:0+18:2+16:0) + TG(18:1+16:1+15:0) + TG(18:1+14:0+17:1) + TG(16:0+18:2+15:0)*	8,277	14,301	-10,942	17,829	0,077
TG(49:3)	*TG(17:1+16:1+16:1) + TG(18:2+15:0+16:1)*	13,17	16,891	-10,925	21,465	0,063
TG(50:0)	*TG(16:0+16:0+18:0)*	0,851	18,145	-20,186	17,664	0,067
TG(50:1)	*TG(16:0+18:1+16:0)*	2,813	14,581	-11,822	11,601	0,068

TG(50:4)	*TG(16:1+18:2+16:1) + TG(16:1+18:3+16:0) + TG(18:1+18:3+14:0) + TG(14:0+18.4+18:0)*	7,741	13,17	-14,835	15,368	0,021
TG(50:5)	*TG(18:3+18:2+14:0)*	0,2	14,277	-18,205	17,076	0,073
TG(51:1)	*TG(16:0+17:0+18:1)*	10,331	23,637	-13,878	17,798	0,049
TG(51:2)	*TG(16:0+17:1+18:1)*	10,323	20,832	-12,158	16,484	0,048
TG(51:3)	*TG(16:0+17:1+18:2) + TG(18:2+18:1+15:0)*	9,477	17,882	-9,88	16,041	0,062
TG(51:4)	*TG(18:2+18:2+15:0) + TG(17:1+18:2+16:1) + TG(17:1+18:3+16:0)*	9,072	18,268	-11,623	15,776	0,045
TG(52:0)	*TG(16:0+18:0+18:0)*	-1,052	17,393	-27,661	20,004	0,037
TG(52:1)	*TG(16:0+18:1+18:0)*	2,836	15,497	-16,254	15,307	0,056
TG(52:5)	*TG(16:0+18:2+18:3)*	6,442	14,558	-12,377	13,388	0,03
TG(53:0)	*TG(17:0+18:0+18:0) + TG(20:0+17:0+16:0)*	9,007	13,712	-7,006	18,917	0,149
TG(53:1)	*TG(18:0+18:1+17:0)*	5,259	16,699	-15,904	19,623	0,076
TG(53:2)	*TG(18:1+18:1+17:0)*	11,218	16,954	-11,578	15,881	0,03
TG(53:3)	*TG(18:2+18:1+17:0) + TG(18:1+18:1+17:1)*	10,835	15,181	-10,778	14,786	0,026
TG(54:0)	*TG(18:0/18:0/18:0)*	-9,384	9,416	-27,752	23,184	0,168
TG(54:1)	*TG(18:0+18:1+18:0)*	3,307	16,198	-22,851	21,7	0,049
TG(54:2)	*TG(18:0+18:1+18:1)*	5,781	15,54	-13,136	16,311	0,069
TG(54:4)	*TG(18:2+18:1+18:1) + TG(18:2+18:2+18:0)*	1,496	9,555	-11,342	11,267	0,066
TG(54:5)	*TG(18:2+18:2+18:1)*	-3,341	13,42	-13,372	8,907	0,095
TG(54:5)	*TG(18:1+20:4+16:0)*	2,431	20,274	-10,084	15,991	0,223
TG(54:6)	*TG(18:2+18:3+18:1)*	-1,02	17,413	-18,33	12,355	0,035
TG(54:6)	*TG(20:4+18:2+16:0)*	2,813	24,352	-12,261	13,924	0,136
TG(54:7)	*TG(18:2+18:3+18:2)*	-3,919	15,732	-21,055	12,559	0,029
TG(54:7)	*TG(20:5+18:2+16:0)*	10,402	12,131	-5,325	29,837	0,261
TG(56:0)	*TG(20:0+18:0+18:0) + TG(22:0+20:0+14:0)*	-12,407	10,331	-23,99	40,052	0,552
TG(56:1)	*TG(22:0+18:1+16:0) + TG(24:0+16:1+16:0)*	-17,526	11,726	-30,301	24,73	0,332
TG(56:2)	*TG(18:1+20:1+18:0)*	-5,945	18,1	-25,985	23,78	0,157
TG(56:3)	*TG(18:1+20:1+18:1)*	-1,187	17,722	-18,642	15,272	0,088
TG(56:5)	*TG(22:5+18:0+16:0) + TG(22:4+18:1+16:0)*	7,35	13,015	-9,604	14,361	0,06
TG(56:5)	*TG(20:4+18:1+18:0)*	19,652	21,938	-0,712	16,649	0,074
TG(56:6)	*TG(22:5+18:1+16:0) + TG(20:4+18:1+18:1)*	8,53	17,951	-6,985	13,28	0,09
TG(56:7)	*TG(22:5+18:2+16:0) + TG(20:4+18:2+18:1)*	7,086	23,972	-10,2	12,676	0,076
TG(56:7)	*TG(22:6+18:1+16:0)*	0,732	12,409	2,505	19,173	0,863
TG(56:8)	*TG(20:5+18:2+18:1) + TG(20:4+18:2+18:2)*	1,663	27,537	-14,09	17,585	0,186
TG(56:8)	*TG(22:6+18:2+16:0)*	18,854	15,372	-1,289	12,085	0,015
TG(58:1)	*TG(24:0+18:1+16:0)*	-13,965	15,863	-31,325	30,75	0,28
TG(58:2)	*TG(18:1+22:0+18:1)*	-14,905	17,198	-33,218	22,472	0,161
TG(58:3)	*TG(22:1+18:1+18:1) + TG(22:1+18:2+18:0)*	-12,815	12,035	-32,512	21,55	0,109
TG(58:4)	*TG(22:0+18:2+18:2)*	-18,12	16,385	-10,366	43,404	0,808
TG(58:6)	*TG(22:5+18:0+18:1)*	11,543	14,322	-6,258	15,301	0,064
TG(58:7)	*TG(22:5+18:2+18:0) + TG(22:4+18:2+18:1) + TG(22:5+18:1+18:1)*	16,319	25,183	-6,345	16,766	0,065
TG(58:8)	*TG(22:5+18:2+18:1) + TG(22:6+18:1+18:1)*	1,362	7,864	-5,224	10,828	0,311
TG(58:9)	*TG(20:4+20:4+18:1) + TG(22:6+18:1+18:2)*	3,502	10,894	-6,685	10,908	0,115
TG(58:10)	*TG(20:5+20:4+18:1) + TG(20:4+20:4+18:2)*	4,826	12,068	-10,123	11,421	0,031
TG(59:1)	*TG(24:0+18:1+17:0)*	-25,199	11,834	0,352	102,746	0,702
TG(60:1)	*TG(24:0+18:0+18:1)*	-6,001	17,033	-33,21	36,967	0,184

TG(60:2)	*TG(24:0+18:1+18:1)*	-9,496	18,767	-30,29	30,593	0,21
TG(60:3)	*TG(24:0+18:1+18:2)*	-21,408	9,393	-33,805	23,439	0,319
TG(42:1)	*TG(12:0+18:1+12:0)*	-18,847	29,301	-28,881	33,284	0,602
TG(53:4)	*TG(18:2+17:1+18:1)*	9,892	17,132	-10,459	14,874	0,04
TG(45:1)	*TG(14:0+15:0+16:1)*	-4,474	8,66	-2,861	54,158	0,963
DG(30:0)	*DG(14:0+16:0+0:0)*	18.771	18.4021	-8.027	33.290	0.153
DG(32:0)	*DG(16:0+16:0+0:0)*	-10.813	13.773	-14.132	14.254	0.691
DG(32:1)	*DG(16:0+16:1+0:0)+ DG (14:0+18:1+0:0)*	6.040	20.064	-11.596	18.920	0.133
DG(32:2)	*DG(14:0+18:2+0:0)*	17.929	14.938	-20.658	21.573	0.005
DG(34:0)	*DG(16:0+18:0+0:0)*	-40.197	12.204	-16.870	32.145	0.186
DG(34:1)	*DG(16:0+18:1+0:0)*	2.493	16.651	-11.944	13.507	0.101
DG(34:2)	*DG(16:0+18:2+0:0)*	2.768	12.312	-13.289	11.825	0.035
DG(36:1)	*DG(18:0+18:1+0:0) + DG(16:0+20:1+0:0)*	4.817	19.816	-17.448	14.355	0.028
DG(36:2)	*DG(18:1+18:1+0:0)*	13.889	18.094	-8.990	12.952	0.015
DG(36:3)	*DG(18:1+18:2+0:0)*	-11.106	7.531	-15.779	17.757	0.623
DG(36:4)	*DG(18:2+18:2+0:0)*	-8.373	10.558	-13.063	20.853	0.677
DG(36:4)	*DG(18:1+18:3+0:0)*	7.168	17.931	0.290	19.977	0.552
DG(36:4)	*DG(16:0+20:4+0:0)*	11.874	39.141	-12.408	17.477	0.099
DG(38:5)	*DG(16:0+22:5+0:0) + DG(18:1+20:4+0:0)*	7.289	24.486	-9.643	13.585	0.098
DG(38:6)	*DG(18:2+20:4+0:0)*	15.912	23.822	-7.829	19.129	0.0615

**% change post-dose (2H) vs pre-dose**	Placebo		PXL770		
	**Mean**	**SD**	**Mean**	**SD**	**T test vs placebo**
Total ceramides	4.026	9.371	-0.699	11.121	0.460
Total sphingomyelins	9.171	18.089	-1.715	7.334	0.097

Supplemental Table III. Changes in Individual Triglyceride (TG) and Diacylglyceride (DG) Species, and in Total Ceramides and Sphingomyelins from Pre-Dose to Post-Dose (2H) on Day 14. Related to Figure 4.

**Albumin (g/dL)**	**PXL 770 Mean (SD) [n]**	**Placebo Mean (SD) [n]**
Baseline PXL770	4.392 (0.210) [13]	4.400 (0.356) [4]
Week 4	3.933 (0.192) [12]	3.975 (0.618) [4]
Change from baseline	-0.450 (0.258) [12]	-0.425 (0.350) [4]
**Protein (g/dL)**	**PXL 770 Mean (SD) [n]**	**Placebo Mean (SD) [n]**
Baseline PXL770	6.977 (0.327) [13]	7.575 (0.411) [4]
Week 4	6.392 (0.264) [12]	6.650 (0.835) [4]
Change from baseline	-0.533 (0.368) [12]	-0.925 (0.650) [4]

Supplemental Table IV. Plasma Albumin and Total Protein levels at Baseline and Week 4 (normal range = 3.5-5.0 g/dL albumin and 6.0-8.3 g/dL total protein). Related to Table 3.
